# The Road to CAR T-Cell Therapies for Pediatric CNS Tumors: Obstacles and New Avenues

**DOI:** 10.3389/fonc.2022.815726

**Published:** 2022-01-27

**Authors:** Ian Burns, William D. Gwynne, Yujin Suk, Stefan Custers, Iqra Chaudhry, Chitra Venugopal, Sheila K. Singh

**Affiliations:** ^1^ Michael G. DeGroote School of Medicine, McMaster University, Hamilton, ON, Canada; ^2^ Department of Surgery, McMaster University, Hamilton, ON, Canada; ^3^ Department of Biochemistry and Biomedical Sciences, Faculty of Health Sciences, McMaster University, Hamilton, ON, Canada

**Keywords:** chimeric antigen receptor T-cell, pediatric brain tumor, immunotherapy, CNS tumor, combinatorial immunotherapy

## Abstract

Pediatric central nervous system (CNS) tumors are the most common solid tumors diagnosed in children and are the leading cause of pediatric cancer-related death. Those who do survive are faced with the long-term adverse effects of the current standard of care treatments of chemotherapy, radiation, and surgery. There is a pressing need for novel therapeutic strategies to treat pediatric CNS tumors more effectively while reducing toxicity – one of these novel modalities is chimeric antigen receptor (CAR) T-cell therapy. Currently approved for use in several hematological malignancies, there are promising pre-clinical and early clinical data that suggest CAR-T cells could transform the treatment of pediatric CNS tumors. There are, however, several challenges that must be overcome to develop safe and effective CAR T-cell therapies for CNS tumors. Herein, we detail these challenges, focusing on those unique to pediatric patients including antigen selection, tumor immunogenicity and toxicity. We also discuss our perspective on future avenues for CAR T-cell therapies and potential combinatorial treatment approaches.

## Introduction

Pediatric central nervous system (CNS) tumors are the most common solid tumors diagnosed in children (1). Despite advances in the molecular characterization of these tumors and the fine-tuning of multimodal therapies, numerous patients experience high rates of tumor recurrence and mortality (2, 3). In fact, CNS tumors are the leading cause of pediatric cancer-related death, recently surpassing leukemia (1, 4). Those who survive face lifelong challenges associated with the standard of care (SoC) treatment, which usually consists of surgery, chemotherapy and/or local or craniospinal irradiation. Chemotherapy leaves patients with off-target organ damage and often neurocognitive deficits (5), and radiation causes debilitating damage to the developing brain (6). Given this persistent mortality and morbidity, there is an urgent need for novel therapies that effectively eradicate CNS tumors in children, providing durable remissions while minimizing treatment-related toxicity.

Recent developments in cancer immunotherapy have unveiled targeted treatment strategies that can prevent tumor recurrence and negate long-term neurotoxic sequalae caused by cytotoxic therapies. Immune checkpoint inhibition with antibodies targeting programmed cell death protein 1 (PD-1) and CTLA4 demonstrates superior efficacy in comparison to the SoC in several cancers (7, 8). In many children with relapsed and treatment-refractory leukemia, treatment with Chimeric Antigen Receptor (CAR) T-cells has led to durable remission (9, 10). CAR T-cells are generated by engineering patient T-cells to express the hybrid CAR protein, which contains an extracellular antibody-like domain that recognizes a cancer-specific antigen and intracellular signalling components that trigger an immune response (11). Each new generation of CAR T-cell has comprised more sophisticated co-stimulatory signalling domains, including CD28 and 4-1BB, and other genetic modifications, such as transgenes for cytokine secretion, to optimize anti-tumor activity (12). Currently, CAR T-cells are approved for use in hematological malignancies including relapsed/refractory non-Hodgkin lymphoma, multiple myeloma, and pediatric relapsed acute lymphoblastic leukemia (13). Solid tumors have emerged as the next frontier for CAR T-cell therapies.

Pre-clinical and early clinical trial data have suggested that CAR T-cells could play an important role in the treatment of pediatric CNS tumors, including medulloblastomas (MB), atypical rhabdoid teratoid tumors (ATRT), high grade gliomas (HGG) and ependymomas (14–16). Many obstacles remain, however, to the successful development of CAR T-cell therapies in pediatric CNS tumors. The paucity of targetable antigens and the unfavourable immunological characteristics of these tumors present unique challenges, and children have unique and poorly understood vulnerabilities to treatment-related toxicities. Herein, we review the major challenges associated with developing CAR T-cell therapies specifically for pediatric CNS tumors and present our perspective on possible avenues for the future development of more effective CAR T-cell and combinatorial immunotherapies.

## Challenges

### Antigen Selection

Whereas adult CNS tumors display an abundance of neoantigens that arise from high mutational burden, there is a marked paucity of neoantigens on pediatric CNS tumors (17, 18). Children are exposed to fewer environmental factors that contribute to DNA damage and the resultant lack of neoantigens presents a unique challenge for pediatric immunotherapy target selection. Target antigens should have tumor-specific (little to no expression in normal cells) or tumor-associated (overexpressed in tumor tissue) expression to spare the developing brain from off-tumor toxicity (19). One promising strategy to overcome the paucity of true neoantigens is to target oncofetal antigens, a class of cell surface markers normally expressed exclusively during prenatal tissue development that can become re-expressed during neoplastic transformation (20). For example, CAR T-cells have been developed to target tumor-specific exons of the oncofetal antigen cerebroglycan GPC2 (21, 22). Alternatively, they can be made to target tumor-specific antigen epitopes. CAR T-cells targeting the epidermal growth factor receptor (EGFR) 806 epitope that is uniquely expressed on the surface of tumor cells can effectively eradicate glioblastoma (GBM) cells while sparing EGFR-expressing human fetal astrocytes (23).

In addition to a reduced neoantigen abundance, there is extensive intratumoral phenotypic heterogeneity among pediatric CNS tumor cells (24). Brain tumor initiating cells (BTICs) are an infrequent subpopulation of tumuor cells CAR T-cell that share properties with normal stem cells, including the capacity for limitless self-renewal and proliferation. BTICs are resistant to chemotherapy (25) and radiation (26) and seed pediatric CNS tumor recurrence and leptomeningeal metastasis (27, 28). The identification of a target that selectively marks BTICs may provide an effective means to eradicate therapy refractory tumor cells, thus delaying or preventing recurrence. Unfortunately, existing BTIC markers amenable to immunotherapy in adult gliomas, such as prominin 1 (PROM1; CD133), are also expressed by human neural stem and progenitor cells (29).

Selection of tumor cells with reduced target antigen expression throughout the course of treatment will also induce temporal heterogeneity. This antigen escape is an impediment to effective CAR T-cell treatment (24, 30). Multivalent CARs are a potential way to improve targeting of tumors with heterogenous antigen expression. Bielamowicz et al. demonstrated improved anti-tumor efficacy in GBM models using trivalent CAR T-cells targeting ephrin A receptor 2, human epidermal growth factor receptor 2 (HER2) and interleukin-13 receptor alpha-2 (IL13Rα2) (30). With the same trivalent design, a significant survival benefit was observed in patient-derived xenograft (PDX) models of MB and ependymoma. Notably, modest expression of HER2 and IL13Rα2 on patient samples in this study suggests additional, more highly expressed targets are needed (15).

Currently, there are a limited number of CAR T-cell clinical trials for children with CNS tumors, all at phase I. Targets include HER2, B7 homolog 3 (B7H3), EGFR806, the disialoganglioside GD2 and IL13Rα2 (**Table 1**).

**Table 1:** Current clinical trials investigating CAR T-cells for pediatric CNS tumors.

NCT#	Target	Tumors	Delivery	Ages eligible (years)	Trial location
04510051	IL13Rα2	IL13Rα2-positive recurrent/refractory CNS tumors	ICV	4-25	City of Hope Medical Centre
04185038	B7H3	DIPG, DMG, recurrent/refractory CNS tumors	IT, ICV	1-26	Seattle Children’s Hospital
03638167	EGFR806	EGFR-positive recurrent/refractory CNS tumors	IT, ICV	1-26	Seattle Children’s Hospital
04099797	GD2	GD2-positive CNS tumors including HGG, DIPG, MB	IV	1-18	Texas Children’s Hospital
04196413	GD2	H3K27M-mutated DIPG or spinal DMG	IV	2-30	Stanford University
03500991	HER2	HER2-positive recurrent/refractory CNS tumors	IT, ICV	1-26	Seattle Children’s Hospital
04903080	HER2	HER2-positive recurrent/refractory ependymoma	IV	1-21	Texas Children’s Hospital
02442297	HER2	HER2-positive recurrent/refractory primary CNS tumors or HER2-positive tumors metastatic to CNS	IT, ICV	≥3	Texas Children’s Hospital

DIPG, Diffuse intrinsic pontine glioma; DMG, diffuse midline glioma; MB, medulloblastoma; ICV, intraventricular; IT, intratumoral; IV, intravenous.

All trials are in Phase 1.

### Delivery

In comparison to hematological malignancies, solid tumors and especially CNS tumors situated behind the blood brain barrier (BBB) present unique physical challenges that hinder effective delivery of CAR T-cells. While peripherally infused CAR T-cells have been found to modestly cross the BBB (31–33), numerous pre-clinical studies evaluating the comparative efficacy of intravenous (IV), intratumoral (IT) and intraventricular (ICV) delivery of CAR T-cells targeting CNS tumors have produced compelling evidence favoring locoregional administration *via* surgically-inserted catheter (IT or ICV). Locoregional delivery is associated with more effective tumor infiltration, improved anti-tumor efficacy, and reduced systemic toxicity (16, 34–36). For example, Theruvath et al. tested B7H3 CAR T-cells against ATRT patient-derived xenografts in mice and showed dramatically more rapid tumor homing and expansion with locoregional delivery, in comparison to the far higher doses of CAR T-cells delivered *via* IV. Additionally, significantly higher levels of systemic inflammatory cytokines were detected upon IV delivery (16). Notably, ICV delivery may be superior to IT in cases of leptomeningeal spread, as CAR T-cells are able to more freely traffic throughout the CNS (34). In current pediatric clinical trials, locoregional delivery is the preferred method (**Table 1**).

### Homing and Persistence

Other important challenges impeding the development of effective CAR T-cells for pediatric CNS tumors include CAR T-cell homing and persistence. To improve homing to tumor sites, CAR T-cells expressing chemokine receptors have been developed (37, 38). Once CAR T-cells reach target sites, they must be capable of exerting an antitumoral response prior to exhaustion. Should exhaustion occur prior to tumor clearance, CAR T-cell efficacy drops dramatically. A recent study found that co-expression of AP1 transcription factor, c-Jun, in CAR T-cells led to an increased capacity for expansion, and diminished terminal differentiation. These exhaustion-resistant CAR T-cells also exhibit a dramatic increase in antitumoral efficacy (39).

Additional strategies to improve CAR T-cell persistence and reduce exhaustion include optimizing T-cell activation and co-stimulation signalling and interfering with molecules that impair T-cell activation (40). For example, CAR T-cells engineered to express pro-inflammatory cytokines such as IL-12 and IL-18 and those with constitutively active IL-15 and IL-7 have increased anti-tumor efficacy and improved persistence in solid tumors (41–44). Particularly in immunologically “cold” pediatric CNS tumors, additional inflammatory cytokine secretion by CAR T-cells could also augment local immune cell activation. This benefit must be balanced with local and systemic toxicity associated with increased cytokine production (40). Finally, issues of CAR-T cell persistence can be addressed by optimizing the timing of their delivery. For example, the use of small, frequent (usually weekly) dosing regimens may help maximize the therapeutic window while minimizing infusion-associated toxicity (37). It is unclear, however, whether frequent CAR T-cell dosing translates to improved anti-tumor efficacy in comparison to infrequent or one-time dosing.

### Toxicity

Cytokine release syndrome (CRS), a systemic inflammatory response following excess cytokine production by endogenous immune cells and/or CAR T-cells, and the toxic encephalopathy known as immune effector cell-associated neurotoxicity syndrome (ICANS) that often follows, are major systemic side effects of CAR T-cell therapies targeting hematological malignancies (45). Relatively little is known regarding these toxicities in the context of CAR T-cells for CNS tumors, especially in pediatrics. Nevertheless, the locoregional delivery strategies currently employed with many CNS-targeting CAR T-cell therapies reduce much of the concern for systemic toxicity, which is known to be a dose-dependent (46) manifestation of the systemic administration and peripheral activation of CAR T-cells (16, 45). This is in keeping with the CRS reported by Goff et al. after IV infusion of only the highest dose of EGFRvIII-targeting CAR T-cells in a GBM patient (47), and that most trials with CAR T-cells targeting CNS tumors have shown few adverse events (48). There is, however, reasonable concern for excess cytokine production leading to local CNS toxicity following locoregional delivery. Promisingly, 3 pediatric patients recently treated with locally-infused CAR T-cells targeting HER2 experienced no dose limiting toxicity while still showing local CNS immune activation (49). Interestingly, CRS and ICANS were not predicted by pre-clinical studies of CD19-targeting CAR T-cells (45) – perhaps similarly unexpected toxicities will emerge through the development of CAR T-cells for CNS tumors.

Given that CAR T-cell dosing, antigen affinity and other design factors remain largely empiric, off-target and particularly on-target/off-tumor toxicity are major concerns. Illustrating this concern, Richman et al. showed that high-affinity CAR T-cells targeting GD2 caused fatal encephalitis after acting on normal brain tissues expressing GD2 in a neuroblastoma mouse model (50). It has also been observed that ICV-administered CAR T-cells migrate effectively into the periphery (16), suggesting that even with locoregional delivery strategies, off-tumor toxicity within the periphery must be considered.

In creating CAR T-cells for the pediatric population, attention must be drawn to the fact that the childhood brain and other tissues are still developing and also have different antigen expression in comparison to adults. This is particularly relevant with CAR T-cells targeting known or potential stem cell antigens. For example, CD133 is expressed on neural stem cells (51) and hematopoietic stem cells (52). Hence, while treatment with CD133-targeting CAR T-cells may be tolerated in adults with GBM, this target may not be appropriate in pediatric patients. Preclinical development of novel targets must ensure proper examination of appropriate control tissues, such as human neural stem cells and fetal tissue arrays, to get insights into potential toxicities. Building inducible control into CAR T-cells provides clinicians with the ability to rapidly regulate CAR T-cell activity during treatment and in case of anticipated or unanticipated toxicities. These include suicide genes such as inducible Caspase 9 and herpes simplex virus tyrosine kinase, and cell surface elimination markers that allow for antibody-mediated control (53).

### Tumor Immune Microenvironment

Tumors comprise a distinct network of tumor cells, immune cells, stromal cells, and extracellular matrix proteins, a spectrum collectively termed the tumor immune microenvironment (TIME). Immunologically “hot” tumors comprise high numbers of tumor-infiltrating lymphocytes (TILs) and increased PD-1 ligand expression, whereas immunologically “cold” tumors have low numbers of TILs and reduced PD-1 expression. Pediatric CNS tumors are immunologically cold due to their low mutational burden and a lack of neoantigen expression (54, 55). Cold tumors respond poorly to immune checkpoint inhibition (56) and are associated with poor clinical outcomes (18, 57). Colder tumors are also less responsive to adoptive T-cell and CAR T-cell therapies (58, 59). In such cases, administered CAR T-cells must be capable of activation and infiltration, where endogenous T-cells are unable to do the same. To overcome the cold TIMEs of pediatric CNS tumors, novel CAR T-cell engineering approaches can be applied to optimize their function in these environments. Potential tools include cytokine switch receptors, which transform an inhibitory signal into a growth-inducing signal, and optimization of CAR T-cell metabolism in the hypoxic and reactive oxygen species-filled microenvironment (40).

In addition to being immunologically cold, there is substantial heterogeneity in the TIME between and among pediatric CNS tumor types. To develop effective immunotherapies, this heterogeneity must be understood and exploited. Grabovska et al. analyzed genome-wide DNA methylation data from >6,000 pediatric CNS tumors – interestingly, the immune infiltrate subgroups that they identified exist independent of molecular subgroup and are predictive of outcomes in multiple pediatric tumor types. They also showed that specific molecular drivers like H3.3G34 mutations in HGG are associated with characteristic immune infiltrates independent of tumor subtype (18). In MB, several studies have shown that Sonic Hedgehog tumors have an increased proportion of T-cells in comparison to other subgroups, rendering them promising candidates for immunotherapy (18, 60). Notably, pediatric midline gliomas are exceptionally immunologically cold and have very low inflammatory cytokine expression (61). In comparison to normal brain tissue, Diffuse Intrinsic Pontine Glioma (DIPG) tumors do not display increased macrophage or T-cell infiltration, or PD1L expression (62).

Looking forward, a deeper understanding of the heterogenous and cold TIMEs of pediatric CNS tumors will allow for the development of novel treatment approaches that help overcome these unfavorable environments. In addition to novel CAR T-cell design, combining CAR T-cells with other immunotherapies or small molecules may allow for the induction of a potent inflammatory response and improve outcomes.

## Combinatorial Therapies

Agents, including small-molecule drugs and other immunotherapies, that can prime CAR T-cells to overcome immunosuppressive effects of tumor cells or those that can convert a cold TIME into a hot TIME may act in combination with CAR T-cell therapies to elicit a more powerful antitumoral response in the pediatric CNS (63). Inhibition of the PD-1/PD-1 ligand immune checkpoint axis, which tumor cells exploit to avoid detection from host immune cells, is a strategy that may enhance the activity of CAR T-cells through increased target engagement (63). The development of small molecules capable of targeting PD-1 have been hindered, however, in part due to the hydrophobic PD-1/PDL-1 interface. The use of cytotoxic/cytolytic agents like cisplatin chemotherapy (64, 65), or oncolytic viruses such as HSV-1 G207 (66), can also enhance the effectiveness of immunotherapy by releasing tumor-associated antigens and cytosolic DNA that promote the conversion of a typically cold pediatric TIME into a hot TIME. The latter presents a potential treatment window of opportunity in pediatric CNS brain tumor patients that are treated with chemoradiotherapy. Researchers have exploited a metabolic vulnerability of immunosuppressive regulatory T-cells (T-reg) to overcome their immunosuppressive nature. Small molecule inhibitors of Indoleamine-pyrrole 2,3-dioxygenase (IDO1) reduce T-reg activity in the TIME and increase immunotherapy efficacy (67).

The capacity for small molecules to be administered systemically, penetrate the BBB, and modulate intracellular targets provides combinatorial immunotherapeutic opportunities for small-molecule agents that monoclonal antibodies and other larger molecules cannot fulfill. Cytotoxic and cytolytic agents also have the potential to greatly enhance the efficacy of CAR T-cell therapies. These combinatorial treatment approaches may be the key to overcoming the challenges presented by solid pediatric CNS tumors.

## Discussion

**Figure 1:**
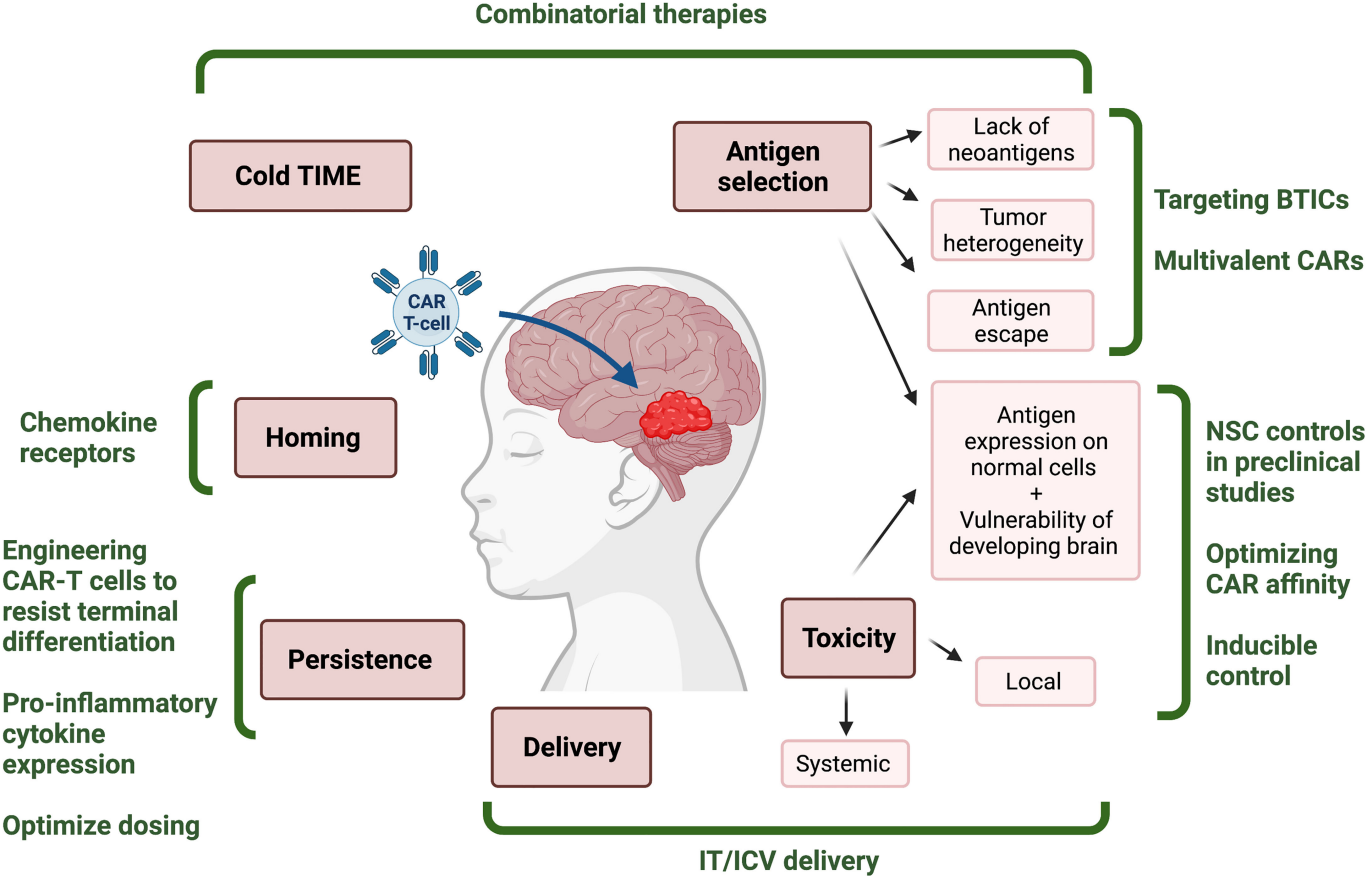
Challenges and potential solutions for development of effective CAR T-cell therapies for pediatric CNS tumors. Infographic depicting the challenges (red) associated with immunotherapies for malignant pediatric CNS tumors, and the proposed solutions (green) that might mitigate them. TIME, tumor immune microenvironment; BTIC, brain tumor initiating cell; NSC, neural stem cell; IT, intrathecal; ICV, intraventricular. Figured created with BioRender.com.

CAR T-cell therapies for hematological malignancies represent major breakthroughs in cancer research and adapting CAR T-cells to target solid tumors represents the next frontier. Here we have reviewed the unique physical and biological challenges associated with developing CAR T-cells for pediatric CNS tumors, and highlighted promising avenues of current and future research (**Figure 1**). The paucity of targetable antigens, intratumoral heterogeneity, and the co-expression of many potential antigens in normal and developing tissues are all fundamental challenges. Potential solutions include using appropriate preclinical controls, exploring BTIC-specific antigens and novel CAR T-cell engineering strategies such as multivalent CARs. In terms of CAR T-cell administration, IT and ICV methods improve delivery and reduce systemic toxicity. There are also many unknowns regarding the local and systemic toxicity of CAR T-cell therapies for pediatric brain tumors and therefore, a cautious approach guided by an awareness of the potential unique susceptibilities of the pediatric brain is called for. It is unclear how treatment of CNS tumors with CAR T-cells may impact brain development. Other novel approaches are also necessary to improve the homing and persistence of administered cells. Finally, the cold and heterogeneous TIMEs of some pediatric CNS tumors necessitate the development and application of novel combinatorial therapies to support CAR T-cells in generating an immune response sufficient to eradicate tumor cells. With creative use of existing and novel therapies and continued innovation in CAR T-cell design, there is potential for a new era of improved outcomes and reduced toxicity for children with CNS tumors.

## Data Availability Statement

The original contributions presented in the study are included in the article/supplementary material. Further inquiries can be directed to the corresponding author.

## Author Contributions

IB, WG, YS, SC, and IC contributed to the conception and drafting of the manuscript. All authors reviewed and approved the final version.

## Conflict of Interest

SS is a shareholder and a scientific advisory board member of Century Therapeutics.

The remaining authors declare that the research was conducted in the absence of any commercial or financial relationships that could be construed as a potential conflict of interest.

## Publisher’s Note

All claims expressed in this article are solely those of the authors and do not necessarily represent those of their affiliated organizations, or those of the publisher, the editors and the reviewers. Any product that may be evaluated in this article, or claim that may be made by its manufacturer, is not guaranteed or endorsed by the publisher.
